# Supplementary material for: Dendritic Cells Internalize Staphylococcus aureus More Efficiently than Staphylococcus epidermidis, but Do Not Differ in Induction of Antigen-Specific T Cell Proliferation
Source: Microorganisms. 2019 Dec 20;8(1):19. doi: 10.3390/microorganisms8010019 (PMC7022728; doi:10.3390/microorganisms8010019)
Supplement: Supplementary file 1 [file microorganisms-08-00019-s001.pdf]

Article – Supplementary data

Dendritic cells internalize *Staphylococcus aureus* more efficiently than *Staphylococcus epidermidis*, but do not differ in induction of antigen-specific T-cell proliferation

Payal P. Balraadjsing [1], Esther C. de Jong [2], Willem J.B. van Wamel [3] and Sebastian A.J. Zaat [1,*]

[1] Dept. of Medical Microbiology, Amsterdam Infection and Immunity Institute, Amsterdam UMC, University of Amsterdam, Amsterdam, The Netherlands
[2] Dept. of Experimental Immunology, Amsterdam Infection and Immunity Institute, Amsterdam UMC, University of Amsterdam, Amsterdam, The Netherlands
[3] Dept. of Medical Microbiology and Infectious Diseases, Erasmus Medical Center, Rotterdam, The Netherlands
* Correspondence: s.a.zaat@amsterdamumc.nl; Tel.: +31-205664863

Supplemental Figures

FIGURE S1

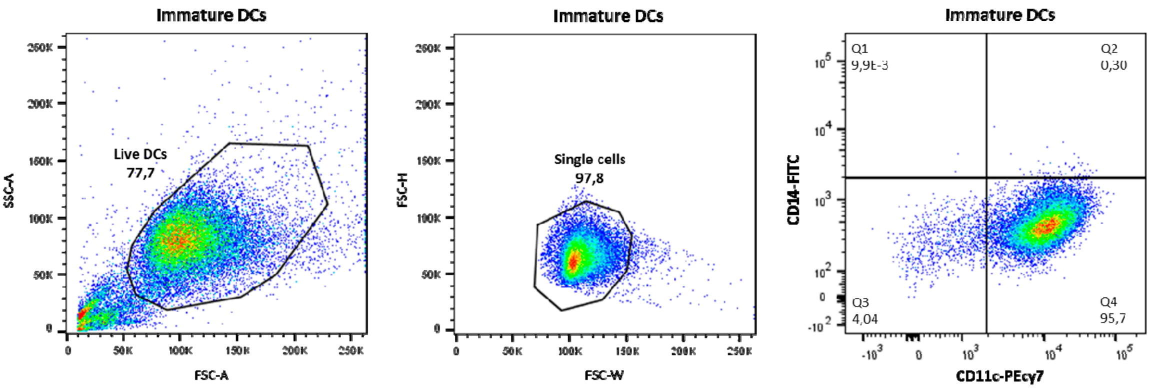

Figure S1: Example of flow cytometry gating and CD11c-positivity of monocyte-derived DCs at day 6 of differentiation. DCs were identified by a combination of forward and sideward scatter properties (left), followed by doublets exclusion (middle) and CD11c-positivity (DC marker) and CD14-negativity (monocyte marker) (right).

FIGURE S2

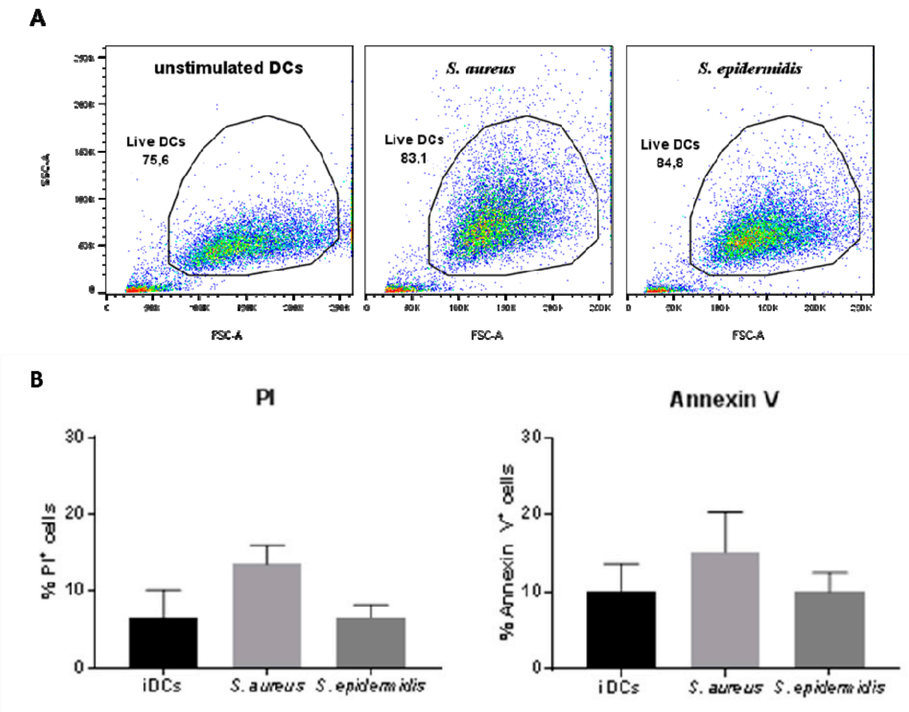

Figure S2: DC cytotoxicity after 48h of infection with *S. aureus* or *S. epidermidis*. (A) Example of flow cytometry gating strategy of live dendritic cells (DCs) based on SSC/FSC dot plot. From left to right; sample with unstimulated, *S. aureus* ATCC49230 stimulated or *S. epidermidis* RP62a stimulated DCs (MOI 100). The FSC and SSC of *S. aureus* or *S. epidermidis* stimulated DCs are very similar to those of unstimulated DCs. DCs stimulated with *S. aureus* or *S. epidermidis* bacteria had a higher SSC since these cells had a higher internal complexity due to phagocytosed bacteria. (B) Cell death and apoptosis of DCs upon stimulation with *S. aureus* ATCC49230 or *S. epidermidis* RP62a (MOI of 100). Propidium iodide (PI) and Annexin V staining was performed to determine cell death and apoptosis by flow cytometry. Non-stimulated DCs (iDCs) were used as controls. The percentages of PI-positive (left) and Annexin-V-positive (right) cells relative to iDCs of three individual donors are indicated as mean + SD.

FIGURE S3

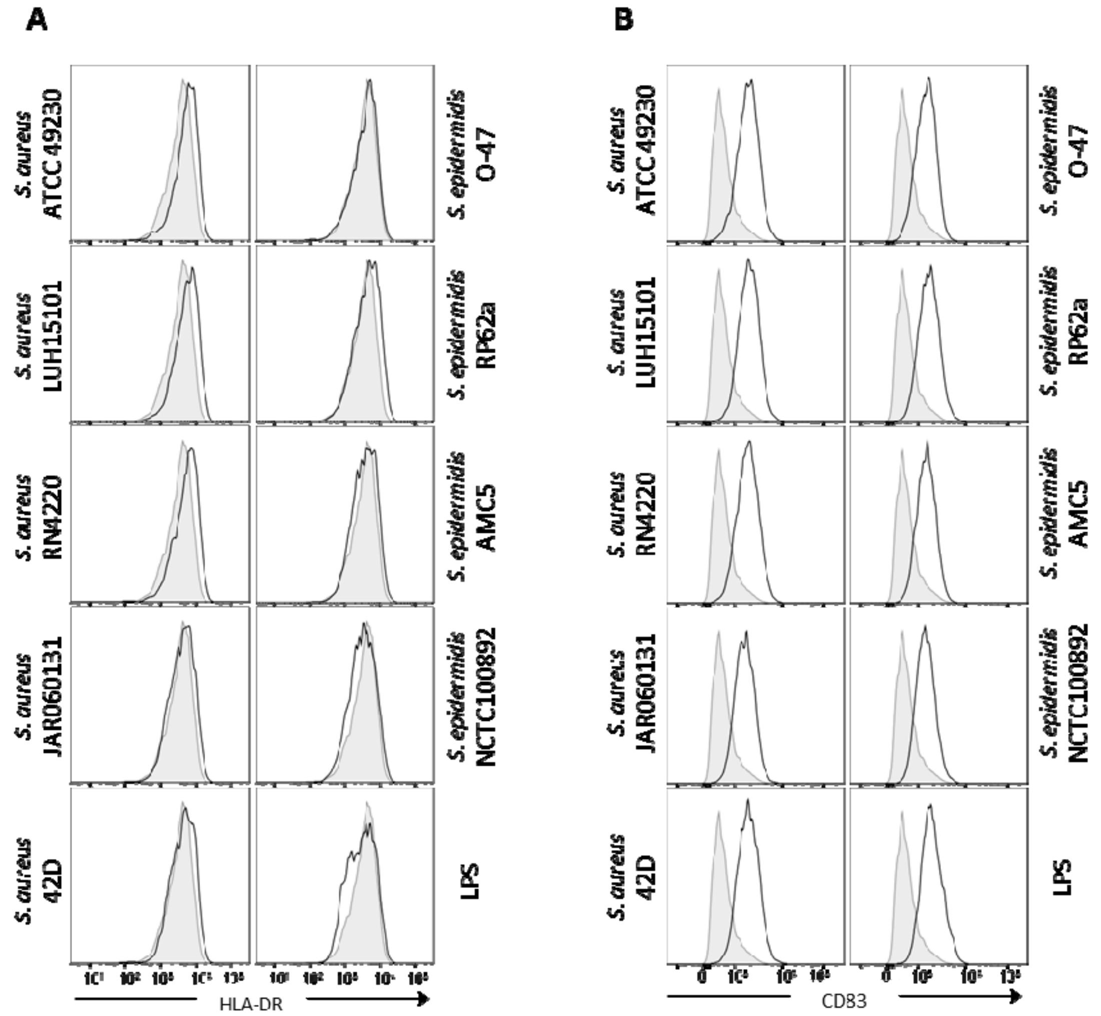

Figure S3: DC maturation marker expression upon *S. aureus* or *S. epidermidis* stimulation. Expression of HLA-DR (A) or CD83 (B) (^{10}log fluorescence intensity) on iDCs (filled) or on DCs stimulated with different strains of *S. aureus* or *S. epidermidis* (solid). Data of 1 experiment out of 2 performed, with similar results.

FIGURE S4

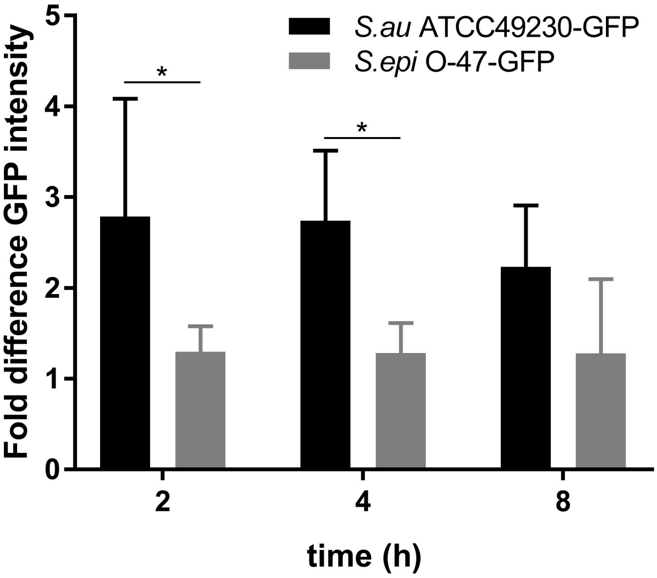

Figure S4: DCs internalize more *S. aureus* than *S. epidermidis* cells. DC uptake of GFP-*S. aureus* ATCC49230 or GFP-*S. epidermidis* O-47 after 2, 4 or 8 h of incubation. Fold difference of geometric mean fluorescence intensity of GFP in DCs which internalized *S. aureus* or *S. epidermidis* relative to unstimulated DCs is shown. The fluorescent intensity of GFP-expressing *S. aureus* ATCC 49230 and *S. epidermidis* O-47 was the same (data not shown). Data are presented as mean + SD of 4 independent experiments, *P<0.05.

FIGURE S5

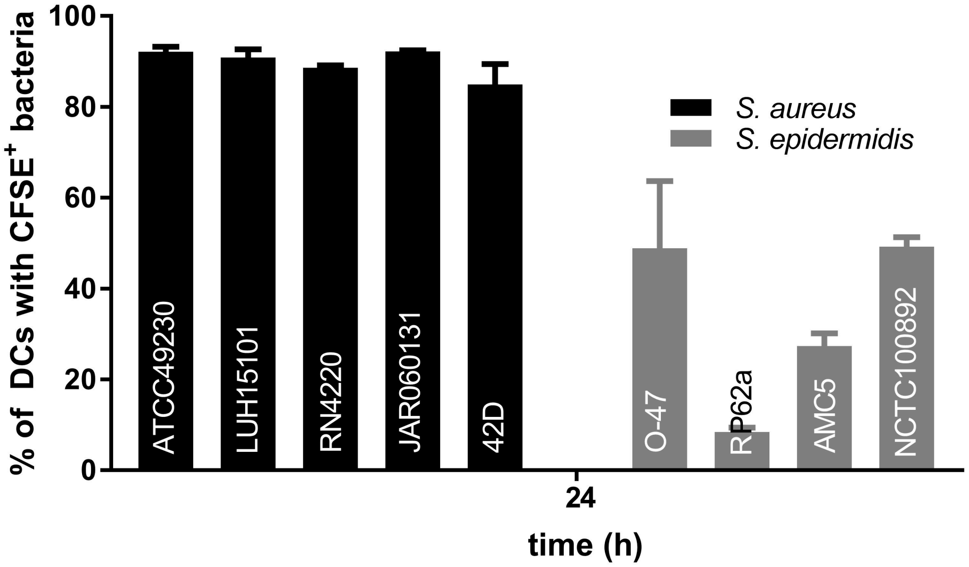

Figure S5: DC uptake of CFSE-labeled strains of *S. aureus* or *S. epidermidis* after 24 h of incubation. The percentage of DCs which internalized fluorescent staphylococci was calculated after flow cytometry. Data are mean + SD of duplicate values.

Supplemental Table

Table S1: Presence of *cap* genes in staphylococci

Strain	Presence of *cap* genes*
S. aureus ATCC 49230	-
S. aureus LUH15101	-
S. aureus RN4220	-
S. aureus JAR060131	-
S. aureus 42D	-
S. epidermidis O-47	+
S. epidermidis RP62a	+
S. epidermidis AMC5	+
S. epidermidis NCTC100892	+

* *capB, capC, capA, capD*
